# SPLACE: A tool to automatically SPLit, Align, and ConcatenatE genes for phylogenomic inference of several organisms

**DOI:** 10.3389/fbinf.2022.1074802

**Published:** 2022-12-08

**Authors:** Renato R. M. Oliveira, Santelmo Vasconcelos, Guilherme Oliveira

**Affiliations:** ^1^ Instituto Tecnológico Vale, Belém, Pará, Brazil; ^2^ Programa de Pós-Graduação em Bioinformática, Instituto de Ciências Biológicas, Universidade Federal de Minas Gerais, Belo Horizonte, Minas Gerais, Brazil

**Keywords:** phylogenomics, pipeline, supermatrix, concatenation, tree

## Abstract

The reconstruction of phylogenomic trees containing multiple genes is best achieved by using a supermatrix. The advent of NGS technology made it easier and cheaper to obtain multiple gene data in one sequencing run. When numerous genes and organisms are used in the phylogenomic analysis, it is difficult to organize all information and manually align the gene sequences to further concatenate them. This study describes SPLACE, a tool to automatically SPLit, Align, and ConcatenatE the genes of all species of interest to generate a supermatrix file, and consequently, a phylogenetic tree, while handling possible missing data. In our findings, SPLACE was the only tool that could automatically align gene sequences and also handle missing data; and, it required only a few minutes to produce a supermatrix FASTA file containing 83 aligned and concatenated genes from the chloroplast genomes of 270 plant species. It is an open-source tool and is publicly available at https://github.com/reinator/splace.

## Introduction

Inferring a species tree using multiple genes is often achieved through two approaches: 1) concatenating aligned genes into a supermatrix or 2) generating a consensus tree (supertree) from separate gene trees. The latter approach looks for congruence among all individual gene trees. The reconstruction of phylogenomic trees containing multiple genes is best achieved by concatenating all aligned genes into a supermatrix. It has greater phylogenetic accuracy because it uses a greater number of sites than examples in which only a single gene is used (Gadagkar et al., 2005). The steady development of next-generation sequencing (NGS) technology makes it easier and cheaper to obtain information from multiple genes from many organisms of interest, resulting in a more robust supermatrix. This supermatrix can then be used in phylogenetic reconstructions to create a species tree.

Many studies have used the supermatrix strategy to infer the phylogeny among species, such as when analyzing genomes from prokaryotic organisms and eukaryotic organelle genomes (mitogenomes and plastomes) (Sims and Kim, 2011). Building a supermatrix can be very time-consuming, especially if there is a large number of genes from many organisms to be used in the analysis. Some published tools, such as SequenceMatrix (Vaidya et al., 2011), TaxMan (Jones and Blaxter, 2006), ScaFoS (Roure et al., 2007), and Phyutility (Smith and Dunn, 2008) aim to concatenate gene files, while others are focused on using the supertree approach, such as TNT (Goloboff and Catalano, 2016), or both supermatrix and supertree methods, such as TREEasy (Mao et al., 2020). Tools that are focused on other strategies, such as NetRax (Lutteropp et al., 2022), which uses the phylogenetic network inference approach, and GeneRax (Morel et al., 2020), which uses the species-tree-aware (STA) approach, were also developed. In this work, we focused on tools that use the supermatrix and supertree strategies.

SequenceMatrix utilizes a graphical user interface (GUI), which may facilitate use by allowing the drag and drop of files with gene alignments in FASTA, TNT, or NEXUS formats. SequenceMatrix can concatenate gene sequences, but it is not able to automatically detect missing data. Its last update was in May 2021, which demonstrates that the software has been continuously maintained since its first release in 2011. As SequenceMatrix was developed in Java, memory consumption may be a problem, particularly if many genes from several organisms are to be analyzed. However, the main limitation that SequenceMatrix presents is that it requires already aligned input files, making the user responsible for detecting missing data in input files. For example, to generate a supermatrix containing 80 genes from 200 organisms, in addition to downloading and organizing the sequences locally, it would also be necessary to group each of the 80 gene sequences across the 200 organisms into 80 different files, align those files separately, and then drag and drop those files into SequenceMatrix, after checking to see if the taxa names were consistent across the 200 sequence entries in the 80 files. This task would be very time consuming and susceptible to errors, leading to delays in the analysis because of the need to review and correct the input information.

TaxMan was developed to facilitate phylogenetic studies by automating sequence acquisition, consensus building, alignment, and taxon selection. It was developed in Perl 5.8.6 and requires a set of prerequisites to be installed in the environment, such as BLAST (Tatusova and Madden, 1999), PostgreSQL (Momjian, 2001), Emboss (Rice et al., 2000), PHRAP (de la Bastide and McCombie, 2007), and POA (Lee et al., 2002). TaxMan accepts GenBank files of the taxa to be analyzed and a file with gene synonyms to be considered, which will be used to extract the gene information automatically from the GenBank files. It cannot automatically detect missing data, so the user is responsible for handling the possible lack of genes. The last TaxMan release was dated September 2006 and it is deprecated to be installed, although the paper is still online.

SCaFoS (Selection, Concatenation, and Fusion of Sequences) is a GUI tool developed in Perl that selects sequences, species, and genes, while dealing with paralogous and xenologous genes, and allows the use of partial genes in the absence of full sequences. It handles missing data by creating chimeric sequences according to the proportion of missing data that the user allows. SCaFoS accepts FASTA, PHYLIP, or Nexus file formats as inputs. The last update of SCaFoS was in October 2007, which means that it no longer has support from the development team. Since the software requires some tools, such as Perl-tk and tree-puzzle, and those libraries evolved through time, it is impossible to execute SCaFoS without a recent code update.

TNT provides a GUI tool for Windows users and command-line tools for Linux and Mac users, allowing the user to run an enormous variety of phylogenetic analyses, simulations, and methods for diagnosing trees and exploring character evolution without automatically handling missing data. The last update of TNT was in October 2022, which means that the tool is still being supported by the developers. The TNT limitation is the same as for SequenceMatrix, i.e., it requires that all the gene files be already aligned, which can be a time-consuming and error-prone activity when performed manually.

Phyutility is a command-line program developed in Java that automates phylogenetic tree, molecular sequence, and alignment manipulation. It accepts FASTA, Newick, and Nexus formats as input to perform tree and sequence manipulation, and it handles missing data by removing or trimming regions of the alignment according to the percentage of missing data allowed by the user. The last update of Phyutility was in September 2012, and it also requires that gene sequences be aligned for use.

TREEasy is the most recent tool to infer phylogeny by concatenating gene sequences. Its last update was in June 2020, allowing options for both GUI and command-line usage. For input, TREEasy needs FASTA files containing the nucleotide sequences of the genes to be included in the analysis and the corresponding amino acid sequence to generate output results for individual gene trees and species trees with supertree and supermatrix approaches. Although TREEasy can automate the alignment of gene sequences using MAFFT, it cannot handle missing data, so the user is responsible for selecting genes shared by all taxa included in the analysis. For the supertree approach, the authors of TREEasy also mention that the tool is only appropriate for working with a few taxa and not hundreds of taxa. Another limitation is that the use of TREEasy requires the installation of eight additional software modules as dependencies, which can be a bottleneck in the analysis if one of the dependencies has an update issue.

Although there is a variety of tools that were developed to aid in phylogenetic/phylogenomic studies, some of them require aligned gene files, which can be a time-consuming and error-prone task if many genes are used, and only a few utilities can handle missing data, although being deprecated. Here we present SPLACE, a tool to automatically SPLit, Align, and ConcatenatE the genes from the species of interest, and generate a supermatrix file and a phylogenetic tree. It can automatically identify and handle missing data, reducing preparation time and the probability of errors in the data to be analyzed. SPLACE can be run with one single command line and is compiled into Docker containers to avoid errors in the installation of dependencies. It is open-source and publicly available at https://github.com/reinator/splace, and its patent is deposited at INPI under the accession #BR512019002834-1. Table 1 summarizes all the tools mentioned previously so that their features can be compared with SPLACE.

**TABLE 1:** Summary of tools developed to aid in phylogenetic/phylogenomic analyses. The approach used by each tool is given in parentheses.

Tool (approach)	Last update	Programming language	S.O.	Dependency	Whether aligns sequences	Whether detects missing data
TaxMan (SM)	September 2006	Perl 5.8.6	Linux	Blast, PostgreeSQL, Emboss, and PHRAP	Yes	No
SCaFoS (SM)	October. 2007	Perl 5.8.0	Linux, Windows XP, and Mac OS X	Perl-tk and tree-puzzle	No	Yes
Phyutility (SM)	September. 2012	Java	Linux, Windows, and Mac OS	Java VM	No	Yes
SequenceMatrix (SM)	May 2021	Java	Linux, Windows, and Mac OS	Java VM	No	No
TNT (ST)	October. 2022	Own language	Linux, Windows, and Mac OS	None	No	No
TREEasy (SM; ST)	July. 2020	Python	Linux, Windows, and Mac OS	MAFFT, IQ-TREE, RAxML-NG, ASTRAL, MP-EST, STELLS2, PhyloNet, and SNaQ	Yes	No
SPLACE (SM)	August. 2022	Python and Bash	Linux, Windows, and Mac OS	Docker	Yes	Yes

SM, supermatrix; ST, supertree.

## Methods

**FIGURE 1:**
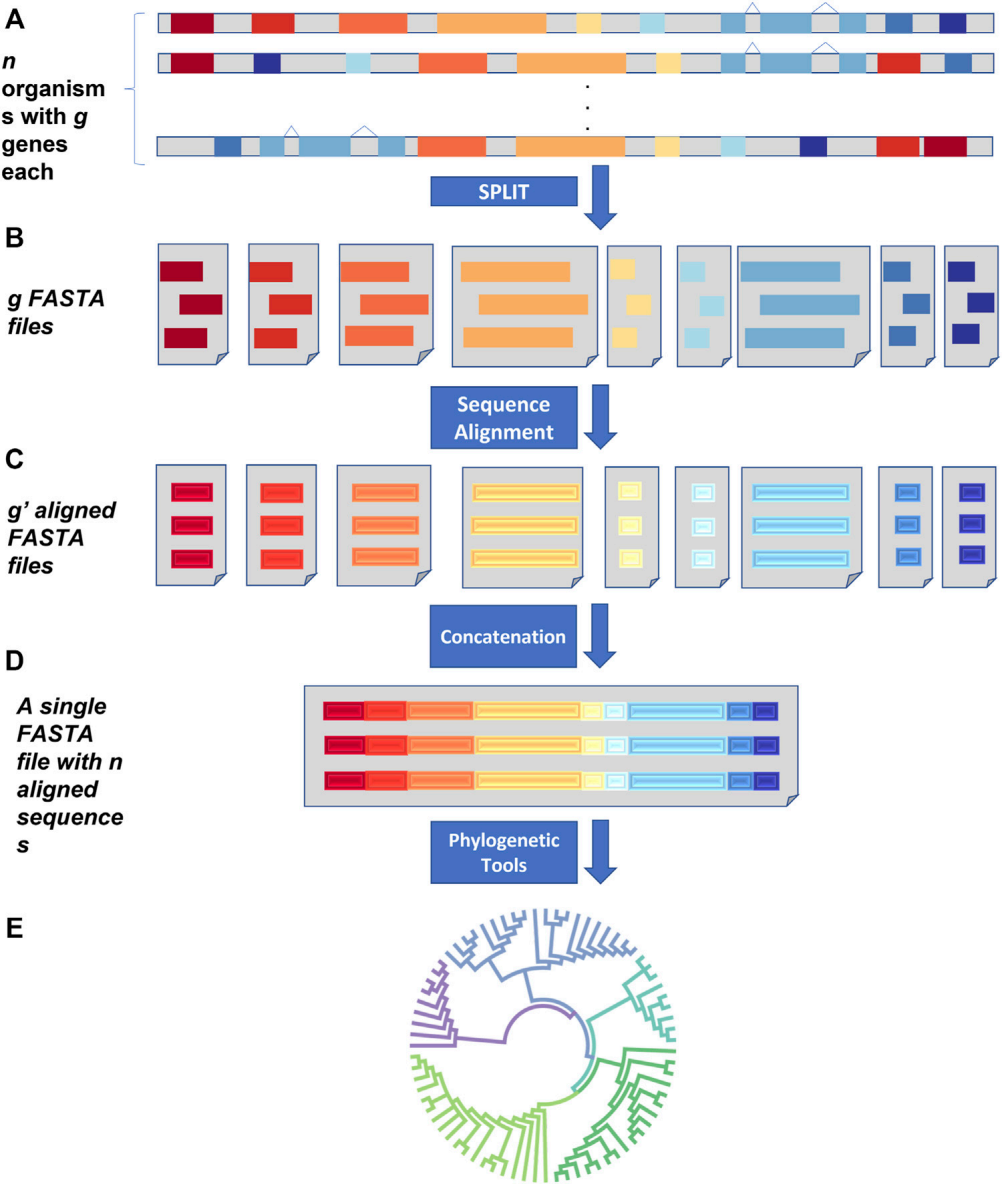
SPLACE workflow. **(A)** SPLACE accepts *n* FASTA files for each organism containing a maximum of *g* genes as input. **(B)** All genes were split into *g* FASTA files containing the same genes from the different organisms and then aligned **(C)**, generating *g´*-aligned FASTA files. **(D)** SPLACE then concatenates the *g´*-aligned genes from the same organism, generating a supermatrix with the resulting *n* sequences, which can then be used to infer phylogeny **(E)**.

For the creation of a supermatrix of *n* organisms, SPLACE will need a text file listing *n* FASTA files, each containing all the *g* genes from a particular organism (Figure 1A). SPLACE can operate in two modes: 1) handling missing data, by specifying a list of genes to consider in the analysis, or 2) considering only the shared genes among the *n* FASTA files of the organisms.

First, SPLACE splits the genes from an organism, gathering genes that have the same name from the *n* organisms into a single FASTA file, generating *g* new FASTA files, each containing the same gene from different organisms (Figure 1B). Then, SPLACE aligns each one of the *g* FASTA files using the MAFFT (Katoh et al., 2002) aligner (with the default parameter –auto), generating *g´* new aligned FASTA files (Figure 1C). Finally, the different genes in the aligned *g´* FASTA files that came from the same organism are concatenated into a single sequence, generating a unique FASTA file with a supermatrix containing *n* sequences, each representing one of the *n* organisms (Figure 1D). The same gene order is followed in all *n* sequences of the supermatrix.

If a list of genes is given as input, SPLACE will be able to handle missing data and if a particular gene is not present in an organism, the space of this missing gene in the concatenated alignment is automatically filled with a “?”, indicating the missing data. If no list of genes is given, the analysis is carried out only with shared genes. Phylogeny can then be reconstructed using the FASTA file with the supermatrix and the method of choice by the user (Figure 1E). SPLACE also generates some reports at the end of the analysis, containing the genes shared among the organisms and a table with the genes found in each organism.

The main limitation of SPLACE is that it requires that the names of the genes be the same in the FASTA files of the different organisms. To facilitate this checking step, the table generated by SPLACE, containing the genes found in each organism may help the researcher determine if there are genes with different names and representations.

## Results and discussion

**FIGURE 2:**
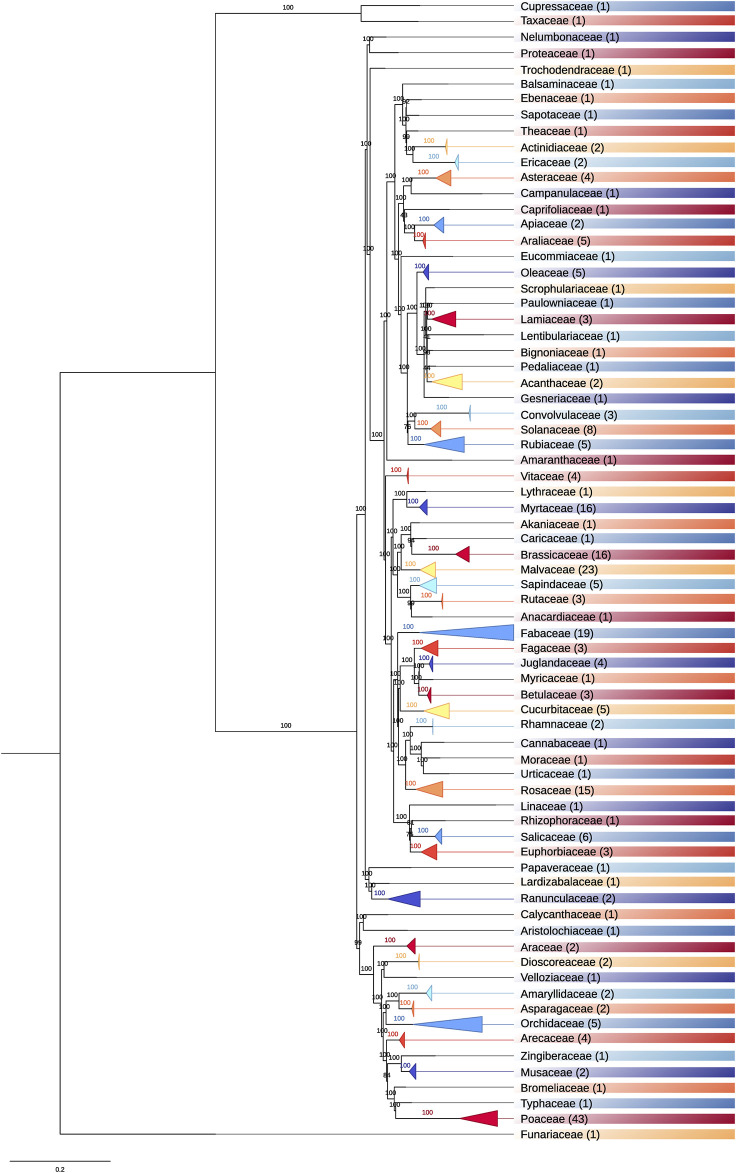
Plastome-based phylogenomic tree for 270 plant species with complete nuclear genomes deposited on NCBI. The number of species a family comprises is given in parentheses. The colors indicate different families.

We intended to benchmark SPLACE with other tools developed to aid in phylogenomic/phylogenetic analysis, but TaxMan, SCaFoS, and Phyutility are obsolete and cannot be installed. SequenceMatrix, TNT, and TREEasy are available for installation, but they are not able to automatically align the sequences or detect missing data. Therefore, we decided not to compare these tools with SPLACE, which is the only utility that can automatically align sequences and detect missing data. Thus, SPLACE was used to build a phylogenomic tree for all plant species with a complete nuclear genome deposited on NCBI (270 species, until April 2022), using their respective chloroplast genes. We downloaded all 270 GenBank files with chloroplast annotation and extracted the gene sequences to compose FASTA files to be used as input. We created a text file containing a list of all FASTA files of the 270 plant species, and we also provided another text file as input containing the 83 genes that we wished to be present in the final results. The supermatrix generated by SPLACE was then submitted to the CIPRES portal (Miller et al., 2010) to generate a maximum likelihood phylogenetic tree using RAxML (Stamatakis, 2014), with the GTRGAMMA model, a bootstrap of 1,000 replicates, and *Physcomitrella patens* (NC_005087) as outgroups. The resulting phylogenomic tree (Figure 2) mainly showed the expected relationships within and among families (Chase et al., 2016), considering the incomplete sampling of taxa due to the selection criteria (species with a complete nuclear genome available). The phylogenomic tree without collapsed branches can be found in Supplementary Figure S1, and all the accessions for the species used and the listed genes are in Supplementary Tables S1, S2, respectively.

The phylogenomic tree represented in Figure 2 comprises 73 families, of which 35 contain more than one species. Poaceae is the family with the most species (43), followed by Malvaceae (23), Fabaceae (19), Brassicaceae (16), Myrtaceae (16), and Rosaceae (15). The remaining families comprise less than eight species. These results show where most efforts are being expended when assembling complete plant nuclear genomes.

SPLACE took only 36 minutes to generate the supermatrix file in a Core i5 2.20 Ghz computer with 12 Gb RAM and using four threads, with a dataset size of 20 MB for the 270 FASTA files containing up to 83 coding genes. If it was necessary to manually group the same genes in FASTA files to further align and manually concatenate them, the time to obtain a supermatrix would be longer. The results show that SPLACE took only a few minutes to extract, align, and generate the phylogeny tree when analyzing many genes from several organisms.

Nowadays, computational clusters provide hundreds of threads and terabytes of RAM memory to run bioinformatics analyses. In this environment, the time taken by SPLACE to generate the supermatrix of 270 plant species could be less than the 36 min required in the previously described computational environment.

## Conclusion

SPLACE is the most recent tool to automatically SPLit, Align, and ConcatenatE gene sequences from several organisms, and also detect missing data. The FASTA files used as input for SPLACE might include either nucleotide or amino acid sequences, since the alignment step with MAFFT automatically recognizes the type of sequence. In addition, the researcher can choose whether the supermatrix generated at the end of the analysis will contain missing data or only shared genes. At the end of the analysis, SPLACE provides a FASTA file containing the supermatrix, which can be used with other utilities, such as tools to select the best evolution model and consequently generate a phylogenomic tree. A table with the genes found in each organism and a table with the shared genes are also generated in the output.

We believe that SPLACE will facilitate phylogenomic analysis by reducing the time needed to separate many genes from several organisms and also reduce the risk of errors.

## Data Availability

The original contributions presented in the study are included in the Supplementary Material, further inquiries can be directed to the corresponding author.

## Abbreviations

NGS, next-generation sequencing; GUI, graphical user Interface; INPI, *Instituto Nacional da Propriedade Industrial*; NCBI, National Center for Biotechnology Information; RAM, random access memory.
