# Supplementary figures and images for: SPLACE: A tool to automatically SPLit, Align, and ConcatenatE genes for phylogenomic inference of several organisms
Source: Front Bioinform. 2022 Dec 8;2:1074802. doi: 10.3389/fbinf.2022.1074802 (PMC9772462; doi:10.3389/fbinf.2022.1074802)

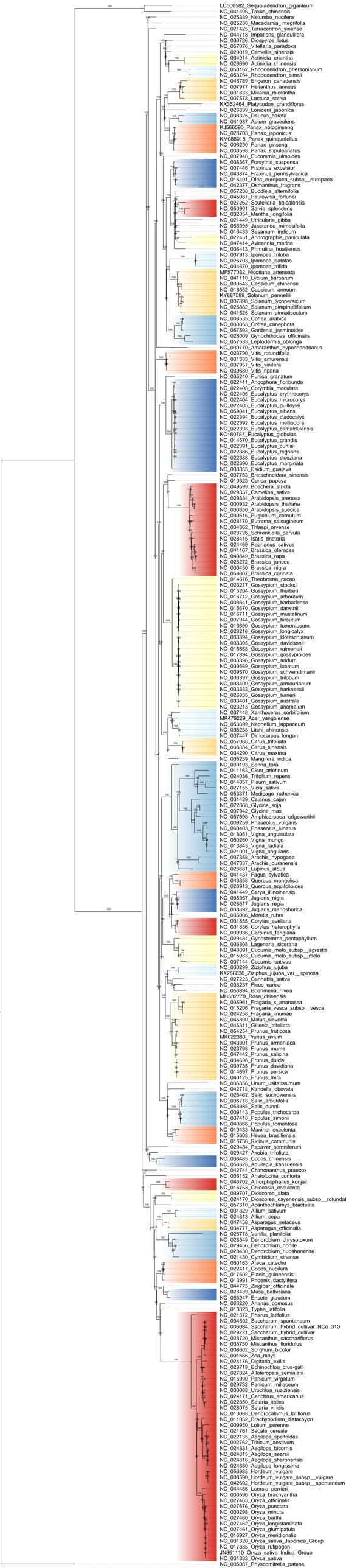

Supplement: Supplementary file 1 [file DataSheet1.PDF]
